# Multimodal workload assessment during simulator-based instrument flight training

**DOI:** 10.3389/fpsyg.2026.1898551

**Published:** 2026-07-14

**Authors:** Jiri Ulvr, Petr Michenka, Vladimir Smrz, Ondrej Mach, Pavlina Engelova, Rostislav Pavlicek

**Affiliations:** Faculty of Military Technology, Air Force Department, University of Defence, Brno, Czechia

**Keywords:** aviation, cognitive workload, flight simulation, NASA-TLX, pilot workload, psychophysiological assessment

## Abstract

**Introduction:**

Pilot performance is influenced by cognitive workload, operational stress, and psychophysiological adaptation during demanding flight tasks. This pilot feasibility study evaluated the applicability of multimodal workload assessment during simulator-based instrument flight operations and explored differences in workload response and operational performance between pilot trainees at different stages of training.

**Methods:**

Twenty-five students from the University of Defence completed a standardized simulator scenario involving an ILS instrument approach under instrument meteorological conditions. Subjective workload was assessed using the NASA Task Load Index (NASA-TLX), physiological response was evaluated using continuous heart-rate monitoring, and operational performance was analyzed using simulator-derived flight parameters. Composite Performance Deviation Index (PDI) and Workload Index (WI) values were subsequently calculated.

**Results:**

The results revealed substantial inter-individual variability in subjective workload, physiological activation, and operational performance. Considerable overlap was observed between year-of-study groups across most evaluated indicators. Several participants maintained relatively stable flight performance despite elevated physiological activation or subjective workload, suggesting that workload response was not uniformly reflected in operational performance measures. The integrated WI-PDI framework additionally provided an exploratory visualization of workload-performance relationships at the individual-participant level.

**Discussion:**

The study demonstrated the practical feasibility of implementing multimodal workload assessment in a simulator-based flight-training environment. The findings support the use of integrated psychophysiological monitoring for future research on workload adaptation and operational performance during pilot training. However, the proposed WI and PDI metrics should be regarded as exploratory measures requiring further validation in larger pilot populations.

## 1 Introduction

Aviation represents one of the most demanding operational environments for human performance. Pilots are required to maintain high levels of cognitive processing, psychomotor coordination and decision-making while operating in dynamic, time-critical and potentially life-threatening conditions. In addition to technical proficiency, successful task execution depends on the pilot's ability to tolerate and regulate multiple forms of workload simultaneously, including psychological, physiological and cognitive stressors (ICAO, 1998; Wickens et al., 2021).

Professional workload in aviation can be understood as the combined effect of mental, physiological and operational demands acting on the individual during task performance. Human performance, however, is inherently limited by biological and cognitive capacity. When operational demands exceed the pilot's adaptive capabilities, performance deterioration may occur, leading to reduced attention, impaired decision-making, fatigue and increased risk of human error (Hart and Staveland, 1988; Campbell and Bagshaw, 2008). Pilots are routinely exposed to demanding psychological and environmental stressors that may negatively influence both mental health and operational performance (The British Psychological Society ed., 2017; Hanakova et al., 2024).

The aviation environment creates a particularly complex form of workload because pilots must continuously process information from multiple sources simultaneously. During flight operations, pilots monitor aircraft parameters, maintain communication with air traffic control, evaluate environmental conditions and anticipate future developments while performing motor tasks under time pressure. Modern cockpit automation further changes the nature of workload rather than eliminating it. Although automated systems reduce certain manual tasks, they often increase monitoring demands and cognitive supervision requirements, particularly during unexpected automation behavior known as “automation surprises” (Parasuraman et al., 1991; Wickens et al., 2021).

From the perspective of human factors research, workload is considered a multidimensional construct rather than a single measurable variable. Hart and Staveland defined workload as the cost required for a human operator to achieve a certain level of performance, emphasizing that workload is associated with the individual rather than solely with the task itself (Hart and Staveland, 1988). This concept formed the theoretical basis for multidimensional workload assessment methods such as the NASA Task Load Index (NASA-TLX), which evaluates mental demand, physical demand, temporal demand, effort, frustration and perceived performance (Hart and Staveland, 1988; NASA, 2010).

In aviation, workload is commonly divided into psychological, physiological and cognitive domains (Němec, 2012). Psychological workload includes emotional stress, responsibility, sustained vigilance and decision-making under pressure (Socha et al., 2020). Pilots may be exposed to significant psychosocial stressors associated with responsibility for human lives, mission success and operational safety (The British Psychological Society ed., 2017; ICAO, 1998). Prolonged psychological overload may negatively influence concentration, situational awareness and emotional stability, eventually contributing to chronic stress or burnout syndrome (Kebza and Šolcová, 2003). Individual responses to operational stressors may vary considerably depending on personal characteristics, coping strategies and resilience-related competencies, which have been identified as important factors influencing adaptation to demanding military environments (Bekesiene et al., 2024; Smaliukienè et al., 2024).

Physiological workload arises from environmental and biomechanical stressors associated with flight operations. Pilots may be exposed to acceleration forces (+Gz), vibration, noise, circadian rhythm disruption and other physiological stressors associated with flight operations. These stressors directly affect cardiovascular, respiratory and neurophysiological functioning and may impair psychomotor performance, visual perception and cognitive efficiency (Campbell and Bagshaw, 2008). Fatigue and sleep deprivation are particularly important operational factors. Studies in military aviation indicate that fatigue contributes to approximately 4–8% of aviation accidents, while a substantial proportion of military pilots report operating aircraft despite severe sleepiness (Wingelaar-Jagt et al., 2021; Switzer and Mendez, 2025).

Cognitive workload reflects the demands placed on working memory, information processing and attentional control during task execution. According to cognitive load theory, total cognitive load consists of intrinsic, extraneous and germane components competing for limited working-memory capacity (Paas et al., 2003; Paas and Van Merriënboer, 1994). Excessive cognitive workload may lead to impaired situational awareness, delayed reactions, non-systematic decision-making and reduced operational effectiveness (Young, 2009; Li et al., 2022).

The importance of human factors in aviation safety has increased despite technological advances. Historical analyses demonstrate that while technical failures have decreased over time, human factors remain the dominant contributor to aviation accidents (Lázaro et al., 2024). Models such as SHELL, Reason's Swiss Cheese Model and HFACS emphasize that pilot performance is influenced not only by the individual operator, but also by interactions between humans, technology, procedures, organizational structures and the operational environment (ICAO, 1998; Shappell and Wiegmann, 2000).

Pilot training therefore increasingly incorporates human factors and workload management principles. Flight simulators provide a controlled and safe environment for inducing operational stress while allowing systematic assessment of pilot responses under varying workload conditions (ICAO, 1998). Simulator-based training enables exposure to realistic operational scenarios without the operational risks associated with real flight operations and has become an important tool for studying pilot workload and performance.

Recent research demonstrated that combining subjective workload measures with physiological indicators improves the assessment of pilot mental workload and operational performance (Hebbar et al., 2021; Wanyan et al., 2014). Because workload is a multidimensional construct, subjective, physiological, and performance-based measures provide complementary information and should ideally be interpreted together rather than separately (Alaimo et al., 2020; Luzzani et al., 2024). Heart rate variability and cardiac activity have shown strong associations with workload fluctuations during simulated flight tasks (Mansikka et al., 2016; Wang et al., 2024). Furthermore, studies using simulated aviation environments confirmed that workload significantly increases during multitasking and complex maneuvers, particularly among less experienced pilots (Li et al., 2022; Zhou et al., 2024; de Almeida et al., 2026).

Despite growing interest in pilot workload assessment, there remains limited research focused specifically on feasibility-oriented evaluation of workload monitoring during simulator-based pilot training in simulator conditions. Understanding the relationship between subjective workload perception, physiological adaptation and operational performance may contribute to safer and more effective training methodologies.

The aim of this pilot feasibility study was therefore to evaluate the applicability of simulator-based workload assessment in pilot trainees and to explore potential differences in workload tolerance and performance between students at different stages of flight training. The study involved students of the University of Defense representing different levels of training experience. Subjective workload, physiological indicators and performance-based outcomes were assessed to provide a comprehensive evaluation of workload-related changes in pilot performance during simulated flight tasks. Understanding workload adaptation during early flight training may contribute to improved pilot selection, training optimization and operational safety. The study also documented protocol implementation, data acquisition procedures, and participant response to the testing environment.

## 2 Materials and methods

The study was designed as a pilot feasibility study evaluating simulator-based workload assessment in pilot trainees. A total of 25 students of the University of Defense participated in the experiment and were divided into 5 year-of-study groups, with five participants in each group, corresponding to the first through fifth academic years of the pilot training programme. The sample consisted of 23 male and 2 female participants. Because of the limited number of female participants, sex-specific analyses were not performed. Participants represented different stages of flight training and completed identical simulator flight tasks under standardized experimental conditions.

Flight experience differed substantially between participants and was not fully represented by year of study alone. Several students entered the programme with previous flight experience and cumulative flight hours varied considerably within some year groups. Therefore, year of study should be interpreted only as an approximate indicator of training progression rather than a direct measure of operational experience. Descriptive flight-experience characteristics are presented in Table 1.

**Table 1:** Flight experience characteristics according to year of study.

Year of study	Previous experience *n* (%)	Total flight hours mean ±SD	PIC hours mean ±SD	IFR hours mean ±SD
1st	2/5 (40%)	0 ± 0	0 ± 0	0 ± 0
2nd	3/5 (60%)	67 ± 84	29 ± 41	0 ± 0
3rd	0/5 (0%)	48.2 ± 0.4	9.0 ± 0.7	1.2 ± 0.4
4th	1/5 (20%)	114 ± 60	32 ± 23	5.4 ± 1.7
5th	0/5 (0%)	109 ± 2	35 ± 0.7	9.0 ± 1.0

Source: Compiled by the authors.

Before the experiment, all participants were familiarized with the simulator environment and experimental procedures and received standardized instructions regarding the flight task, operational objectives and assessment procedures. Participation was voluntary and written informed consent was obtained from all participants prior to testing. The studies involving humans were reviewed and approved by the Ethics Committee of the University of Defense, Brno, Czech Republic, on 25 July 2025. No separate approval or protocol identification number was assigned by the Ethics Committee. The study was conducted in accordance with institutional requirements, the Declaration of Helsinki, and applicable ethical standards.

### 2.1 Experimental environment

The experiment was conducted using the flight simulation facilities of the University of Defense. The simulator environment was based on X-Plane 12 configured for instrument flight operations. The simulated aircraft was a Cessna 172SP equipped with Garmin G1000 avionics and mechanical flight controls. The simulator enabled continuous acquisition of flight-performance data with a sampling frequency of 5 Hz. Recorded parameters included altitude, Indicated Airspeed (IAS) and vertical speed.

Simulator-based flight assessment represents a widely accepted method in aviation human factors research because it enables standardized and repeatable exposure to operational workload under controlled conditions (ICAO, 1998; Wei et al., 2014).

Environmental conditions, aircraft configuration and operational procedures were standardized for all participants in order to minimize external variability and ensure comparability between experimental sessions.

### 2.2 Flight scenario

The experimental task was based on the ILS Y RWY 27 (Instrument Landing System) instrument approach procedure at Pardubice Airport. The scenario was intentionally selected to induce increased cognitive and operational workload under instrument meteorological conditions while remaining feasible for participants without completed IFR qualification.

Meteorological conditions were manually configured using an IFR (Instrument Flight Rules) Precision profile in X-Plane 12. The cloud layer was set to overcast conditions (8/8) with a cloud base at ~950 ft MSL and cloud tops around 3200 ft MSL. Wind conditions were set from 180° at 15 kt with gusts up to 20 kt. Runway visual range was configured to 550 m without precipitation. Air temperature was 14 °C and atmospheric pressure was set to 1013 hPa. These conditions were identical for all participants.

The flight began southwest of the initial approach fix at an altitude of 3,000 ft and heading 025°. The autopilot initially guided the aircraft toward the IAF (Initial Approach Fix). Data recording started before reaching the IAF, while performance evaluation started after passing the IAF.

Participants subsequently performed:

- a climb to 4,000 ft with a target vertical speed of approximately 850 ft/min;
- level flight at 4,000 ft while maintaining 100 kt IAS;
- descent to 3,000 ft with a target vertical speed of approximately −850 ft/min;
- and final ILS approach tracking using the localizer frequency 109.350 MHz.

From the final approach fix, participants followed the 3° glide path while maintaining approximately 100 kt IAS. During the final landing configuration phase, participants reduced airspeed to 85 kt. The total scenario duration was approximately 20 min.

The experimental scenario was designed to induce varying levels of cognitive workload through continuous instrument monitoring, procedural task execution, navigation management, aircraft control, decision-making under time pressure and simultaneous management of multiple operational tasks.

### 2.3 Subjective workload assessment

Subjective workload was evaluated using the NASA Task Load Index (NASA-TLX), one of the most frequently used multidimensional workload assessment methods in aviation psychology and human factors research (Hart and Staveland, 1988).

The NASA-TLX evaluates perceived workload across six dimensions:

- Mental Demand;
- Physical Demand;
- Temporal Demand;
- Performance;
- Effort;
- Frustration.

Participants completed the questionnaire immediately after the simulator task using standardized NASA-TLX procedures. Both the Raw Task Load Index (RTLX) and the Weighted Workload Level (WWL) scores were calculated and subsequently used for workload analysis. The NASA-TLX method was selected due to its extensive validation in simulator-based aviation studies (Hart and Staveland, 1988; Hebbar et al., 2021).


$$\begin{array}{c} WWL=\frac{\sum_{i=1}^{6}\left(R_{i}\times W_{i}\right)}{15}\\ RTLX=\frac{\sum_{i=1}^{6}R_{i}}{6} \end{array}$$

where:

- *R*_*i*_ represents the rating score;
- *W*_*i*_ represents the weighting coefficient.

### 2.4 Physiological measurements

Physiological workload indicators were obtained using continuous heart-rate monitoring throughout the simulator session. Heart rate was measured using the Polar H10 chest strap sensor based on electrocardiographic measurement principles. The sensor was connected via Bluetooth to the Polar Flow mobile application, enabling continuous recording and export of physiological data. The recorded data were subsequently processed in MATLAB.

Cardiovascular measures are commonly used in aviation medicine and human-factors research because physiological activation is closely associated with stress and mental workload during demanding flight operations (Mansikka et al., 2016; Wang et al., 2024; Luzzani et al., 2024). A recent review identified cardiac activity as the most frequently used physiological domain for workload assessment in both simulator-based and real-flight environments, reflecting its sensitivity to workload-related psychophysiological changes and its practical applicability in operational settings (Masi et al., 2023). Although heart-rate variability (HRV) provides detailed information regarding autonomic regulation, heart-rate measures remain widely used indicators of psychophysiological activation in aviation studies (Mansikka et al., 2016; Masi et al., 2023). In addition, recent evidence suggests that HRV responses may vary considerably according to age, flight experience, task characteristics, and flight phase, which can complicate comparisons between studies and operational contexts (Wang et al., 2024).

The primary objective of the present pilot feasibility study was not to provide a comprehensive assessment of autonomic regulation, but rather to evaluate the practical applicability of continuous physiological monitoring during simulator-based flight training and to compare physiological responses across relatively short flight segments. Under such conditions, heart-rate measures provide a practical and readily interpretable indicator of cardiovascular activation associated with workload and operational stress. The choice of HR was additionally motivated by the feasibility-oriented nature of the study, where continuous monitoring with minimal instrumentation burden and straightforward interpretation of physiological responses were considered advantageous (Masi et al., 2023).

Before the simulator scenario, resting heart rate (*HR*_*rest*_) was obtained during a 5-min baseline period while participants were seated in the simulator cockpit standardized resting conditions. Participants were instructed to remain seated, avoid unnecessary movement, and breathe normally. This individual baseline value was subsequently used for normalization of cardiovascular responses during task performance. Heart-rate data were exported from the Polar Flow application and processed in MATLAB for subsequent analysis.

The following physiological parameters were evaluated:

- average heart rate (*HR*_*mean*_);
- maximum heart rate (*HR*_*max*_);
- relative heart-rate increase (Δ*HR*);
- percentage of time above workload threshold (*T*_*HR*>*thr*_);
- physiological workload score (*S*_*HR*_).

The relative increase in heart rate was calculated as:


$$\begin{array}{c} \text{Δ}HR\left[\%\right]=\frac{{HR}_{task}-{HR}_{rest}}{{HR}_{rest}}\times 100 \end{array}$$

The workload threshold was defined as:


$$\begin{array}{c} {HR}_{thr}={HR}_{rest}\times 1,3 \end{array}$$

The workload threshold was therefore defined as 130% of the individual resting heart rate. This relative threshold was selected as an individualized workload-activation criterion intended to account for inter-individual differences in baseline heart rate and to identify periods of elevated cardiovascular activation relative to each participant's resting state. Because no universally accepted heart-rate threshold for pilot workload assessment currently exists, the selected threshold should be interpreted as an exploratory and pragmatic criterion rather than a validated physiological cut-off (Wang et al., 2024).

The percentage of time above the threshold was calculated as:


$$\begin{array}{c} T_{HR>thr}\left[\%\right]=\frac{t\left(HR>{HR}_{thr}\right)}{t_{total}}\times 100 \end{array}$$

Where *t(HR*>*HR*_*thr*_*)* represents the cumulative duration during which heart rate exceeded the predefined threshold and *t*_*total*_ represents the total duration of the analyzed flight segment.

To enable integration of physiological variables expressed on different scales, Δ*HR* and *T*_*HR*>*thr*_were transformed into standardized 0–100-point scores using min–max normalization within the study sample:


$$\begin{array}{c} S_{\text{Δ}HR}=\frac{\text{Δ}{HR}_{i}-\text{Δ}{HR}_{\min}}{\text{Δ}{HR}_{\max}-\text{Δ}{HR}_{\min}}\times 100\\ S_{T>thr}=\frac{T_{i}-T_{\min}}{T_{\max}-T_{\min}}\times 100 \end{array}$$

Higher values indicated greater cardiovascular activation.

The final physiological workload score was calculated as:


$$\begin{array}{c} S_{HR}=\frac{S_{\text{Δ}HR}+S_{T>thr}}{2} \end{array}$$

Equal weighting of both components was applied because no empirical evidence was available to justify differential weighting within the scope of this pilot feasibility study. Consequently, SHR should be regarded as an exploratory composite indicator developed for comparative assessment of physiological workload rather than as a validated psychophysiological metric.

The SHR metric was designed to combine both the intensity of cardiovascular activation (Δ*HR*) and the duration of elevated activation (time above threshold). Higher *S*_*HR*_ values therefore indicate greater cardiovascular activation resulting from either stronger heart-rate elevation, longer duration above the predefined threshold, or a combination of both.

### 2.5 Flight performance assessment

Operational performance was evaluated using deviations from predefined flight parameters recorded during the simulator session. Performance-based indicators provide objective information regarding the behavioral consequences of increased workload and operational stress (Li et al., 2022; Zhou et al., 2024).

The following parameters were analyzed:

- altitude deviation;
- airspeed deviation;
- vertical speed deviation.

The flight was divided into five evaluated segments corresponding to:

- climb;
- level flight at 4,000 ft;
- descent;
- level flight at 3,000 ft;
- ILS approach.

For each segment, the percentage of time outside the tolerance range and the root mean square error relative to the target flight profile were calculated. Higher deviation values represented lower operational precision.

### 2.6 Performance deviation index (PDI)

To enable integrated evaluation of operational performance across all evaluated flight segments, a composite PDI was developed.

For each flight segment, two performance characteristics were evaluated:

- percentage of time outside the prescribed tolerance limits;
- root mean square error (RMSE) relative to the target flight profile.

Because these indicators were expressed in different units and scales, both measures were transformed into standardized 0–100-point scores using min–max normalization within the study sample. For each parameter, higher scores indicated greater deviation from the prescribed flight profile and therefore lower operational performance.

The normalized deviation score was calculated as:


$$\begin{array}{c} S_{out}=\frac{x_{out,i}-x_{out,\min}}{x_{out,\max}-x_{out,\min}}\times 100\; \end{array}$$

where:

- *x*_*out, i*_ represents the percentage of time outside the tolerance limits for participant i;
- *x*_*out, min*_ and *x*_*out, max*_ represent the minimum and maximum observed values within the study sample.

The normalized RMSE score was calculated as:


$$\begin{array}{c} S_{RMSE}=\frac{x_{RMSE,i}\;-\;x_{RMSE,\min}}{x_{RMSE,\max}\;-\;x_{RMSE,\min}}\times 100\; \end{array}$$

where:

- *x*_*RMSE, i*_ represents the RMSE value for participant *i*;
- *x*_*RMSE, min*_ and *x*_*RMSE, max*_ represent the minimum and maximum observed RMSE values within the study sample.

The final score for each flight-performance component was obtained as the arithmetic mean of both normalized indicators:


$$\begin{array}{c} S_{segment}=\frac{S_{out}+S_{RMSE}}{2} \end{array}$$

This procedure was applied separately to:

- airspeed control (*S*_*IAS*_);
- climb vertical-speed control (*S*_*VS*1_);
- altitude-hold performance at 4,000 ft (*S*_*ALT*1_);
- descent vertical-speed control (*S*_*VS*2_);
- altitude-hold performance at 3,000 ft (*S*_*ALT*2_);
- ILS approach performance (*S*_*APP*_).

The composite Performance Deviation Index was then calculated as:


$$\begin{array}{c} PDI=\frac{S_{IAS}+S_{ALT1}+S_{ALT2}+S_{VS1}+S_{VS2}+S_{APP}}{6} \end{array}$$

where:

- *S*_*IAS*_ represents airspeed-control deviation;
- *S*_*ALT*1_ and *S*_*ALT*2_ represent altitude -hold deviation during level-flight segments;
- *S*_*VS*1_ and *S*_*VS*2_ represent vertical-speed deviation during climb and descent;
- *S*_*APP*_ represents deviation from the ideal ILS approach trajectory.

Equal weighting of all six component scores was applied because no empirical evidence was available to justify differential weighting of individual flight segments within the scope of this pilot feasibility study. The objective was to obtain a simple integrated indicator describing overall deviation from the prescribed flight profile across the complete simulator scenario.

Higher PDI values indicate greater deviation from the prescribed flight profile and therefore lower overall flight-performance accuracy. Consequently, the PDI should be regarded as an exploratory composite metric developed for comparative assessment of simulator performance rather than as a validated operational-performance index. Formal validation of the weighting structure, normalization procedure, and alternative aggregation approaches should be addressed in future studies involving larger samples.

### 2.7 Workload index (WI)

To evaluate psychophysiological workload, a Workload Index (WI) was calculated separately from operational performance indicators.

The WI combined:

- physiological workload score (*S*_*HR*_);
- weighted NASA-TLX score (*WWL)*.

Both components were expressed on a common 0–100 scale prior to aggregation, allowing equal contribution of physiological and subjective workload indicators.

The index was defined as:


$$\begin{array}{c} WI=\frac{S_{HR}+WWL}{2} \end{array}$$

The subjective workload component was represented by the weighted NASA-TLX score (WWL), calculated according to the standard NASA-TLX weighting procedure described above. The WI was intended to provide a descriptive composite representation of psychophysiological workload experienced during the simulator task independently of operational flight performance. Higher WI values indicate greater combined subjective and physiological workload.

Because the index was developed specifically for the purposes of this pilot feasibility study and has not undergone formal validation, WI should be interpreted as an exploratory composite measure intended for comparative assessment within the present sample rather than as a validated workload metric. Future studies should evaluate alternative weighting approaches, construct validity, reliability, and sensitivity of the index in larger pilot populations.

### 2.8 Data processing and descriptive analysis

All collected data were processed following completion of the experimental sessions.

Descriptive statistical methods were used to evaluate the central tendency, variability and distribution characteristics of the measured parameters. Comparisons between year-of-study groups were performed to explore differences in workload response and operational performance across different stages of pilot training.

Because of the pilot feasibility design and the small number of participants within each year-of-study group (*n* = 5), inferential analyses were considered exploratory. In addition to descriptive statistics, non-parametric Kruskal–Wallis tests were performed to explore potential differences between year-of-study groups. Effect sizes were estimated using epsilon-squared (ε^2^). Given the limited sample size, the analyses were intended to identify preliminary patterns and generate hypotheses for future research rather than provide definitive evidence of group differences.

## 3 Results

All 25 participants successfully completed the assigned simulator scenario under standardized instrument meteorological conditions. Feasibility indicators included a 100% task-completion rate, successful acquisition of simulator-performance, physiological, and NASA-TLX data from all participants, absence of major technical failures during data collection, and successful completion of the approximately 20-min protocol by all participants without premature termination. The experimental protocol enabled continuous acquisition of physiological, subjective, and performance-related data throughout all phases of the flight task, supporting the practical feasibility of the proposed multimodal workload assessment methodology in a simulator-based flight-training environment.

The results obtained revealed variability in both operational performance and workload-related indicators across year of study groups. Variability was observed not only between years of study but also among students within the same year-of-study group, indicating substantial inter-individual differences in workload responses and operational performance. Given the pilot feasibility design and limited sample size, the reported findings should be interpreted as descriptive observations rather than statistically confirmed differences between training stages.

### 3.1 Flight performance assessment

#### 3.1.1 Airspeed control performance

Performance in maintaining the required IAS of 100 ± 5 kt was evaluated during the period from stabilization at 4,000 ft until completion of the fifth flight segment. The climb phase was excluded from the analysis because the task objective during this phase focused primarily on vertical-speed control rather than stabilized airspeed maintenance.

Airspeed-control performance was assessed using two complementary indicators:

- the percentage of time spent outside the prescribed tolerance range;
- the root mean square error (RMSE) relative to the target IAS of 100 kt.

To enable aggregation of variables expressed in different units, both indicators were transformed into standardized 0–100-point scores using the min–max normalization procedure described in Section 2.6. The resulting airspeed-control score (*S*_*IAS*_) was calculated as the arithmetic mean of both normalized indicators. Lower *S*_*IAS*_ values indicate better airspeed-control performance.

Equal weighting of both components was applied because no empirical evidence was available to justify differential weighting within the scope of this pilot feasibility study. Consequently, *S*_*IAS*_ should be regarded as an exploratory composite indicator developed for comparative assessment of airspeed-control performance rather than as a validated operational-performance metric.

Descriptive statistics for airspeed-control performance according to year of study are presented in Table 2. Mean *S*_*IAS*_ values ranged from 22.7 ± 10.3 points in fifth-year students to 69.0 ± 24.3 points in first-year students. Considerable within-group variability was observed in several year groups, particularly for the percentage of time spent outside the prescribed airspeed tolerance range and the resulting SIAS values. Inspection of individual observations revealed substantial overlap between year groups despite differences in group means.

**Table 2:** Airspeed -control performance according to year of study [mean ± SD (min–max)].

Year of study	Time outside tolerance [%] mean ±SD (min–max)	IAS RMSE [kt] mean ±SD (min–max)	S_IAS_ [points] mean ±SD (min–max)
1st year	47.61 ± 11.40 (29.42–58.45)	8.63 ± 2.58 (5.37–12.21)	68.97 ± 24.30 (34.28–95.36)
2nd year	30.40 ± 18.12 (12.11–59.85)	6.02 ± 3.22 (3.21–11.41)	38.58 ± 34.23 (6.50–95.56)
3rd year	40.71 ± 10.06 (28.86–52.67)	6.51 ± 1.03 (5.27–7.53)	50.31 ± 15.56 (33.21–67.48)
4th year	25.23 ± 20.86 (5.05–48.13)	5.39 ± 1.62 (3.20–7.06)	30.54 ± 28.31 (0.00–60.73)
5th year	20.28 ± 6.18 (12.94–28.67)	4.78 ± 1.03 (4.00–6.40)	22.66 ± 10.31 (11.69–39.31)

Source: Compiled by the authors.

Exploratory Kruskal–Wallis analysis indicated that differences between year-of-study groups approached statistical significance (*H* = 9.36, *p* = 0.053, ε^2^ = 0.268, Table 3), although the overall effect did not reach the predefined significance threshold.

**Table 3:** Exploratory Kruskal–Wallis comparisons between year-of-study groups.

Variable	H	*p*-value	ε^2^
S_IAS_	9.36	0.053	0.268
S_VS1_	6.67	0.154	0.133
S_ALT1_	2.92	0.572	0.000
S_VS2_	10.25	0.036	0.312
S_ALT2_	10.57	0.032	0.328
S_APP_	2.30	0.680	0.000
PDI	9.19	0.057	0.259
WI	5.22	0.266	0.061
SHR	6.25	0.181	0.113
WWL	3.94	0.414	0.000

Source: Compiled by the authors.

**Figure 1:**
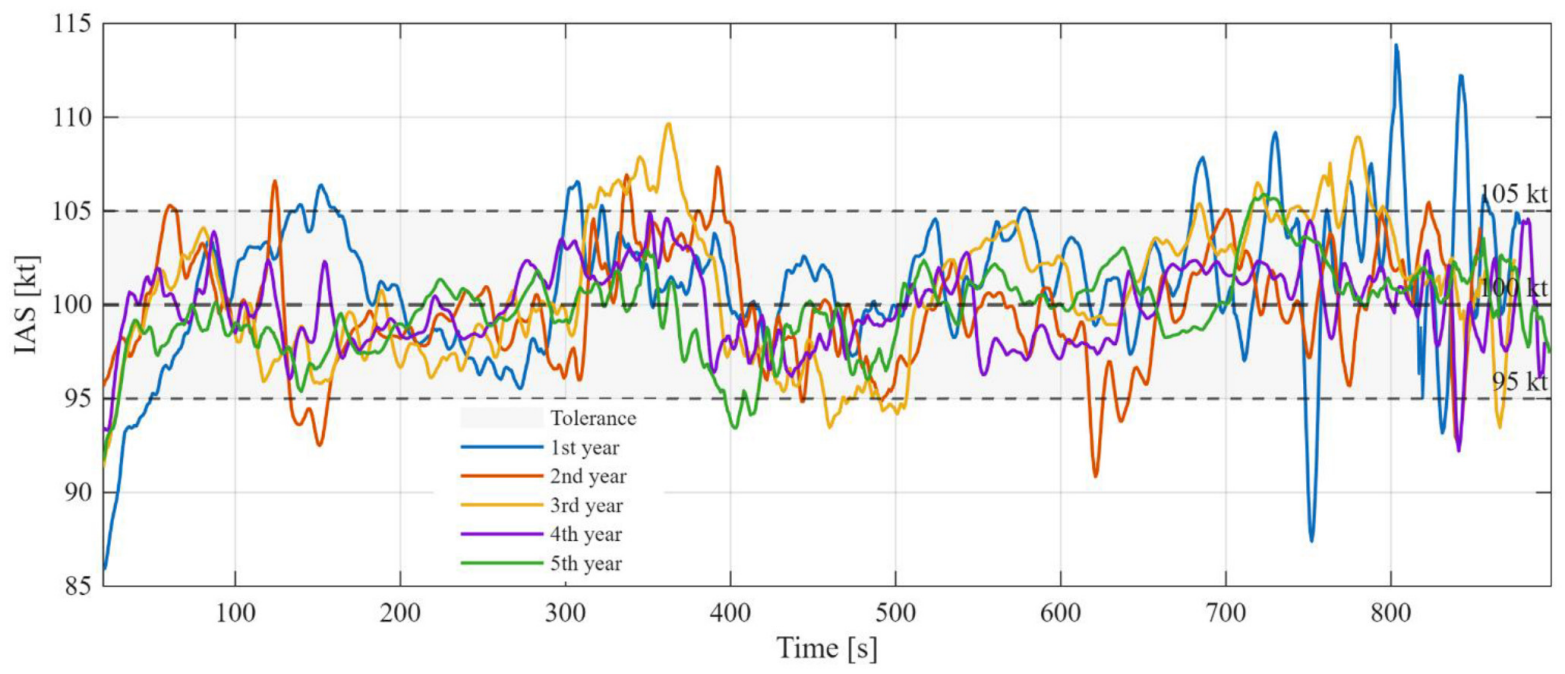
Mean IAS deviations according to year of study. Source: Compiled by the authors.

The temporal progression of IAS throughout the evaluated flight task is shown in Figure 1. Visual inspection suggests differences in airspeed-control behavior between participants and year-of-study groups. However, individual trajectories remained highly variable and no consistent pattern was apparent across all year groups.

#### 3.1.2 Vertical speed performance during climb

The first evaluated flight segment corresponded to the climb phase from the initial approach fix to 4,000 ft. The reference vertical speed was defined as 850 ± 100 ft/min. Segment performance was evaluated using the percentage of time outside the tolerance range and RMSE relative to the reference vertical speed. To enable aggregation of indicators expressed in different units, both variables were transformed into standardized 0–100-point scores using the min–max normalization procedure described in Section 2.6. The resulting climb-performance score (*S*_*VS*1_) was calculated as the arithmetic mean of both normalized indicators. Lower *S*_*VS*1_ values indicate better vertical-speed control performance.

Equal weighting of both components was applied because no empirical evidence was available to support differential weighting of the two performance indicators. Consequently, *S*_*VS*1_ should be considered an exploratory composite indicator developed for comparative assessment of climb performance rather than as a validated operational-performance metric.

Descriptive statistics for climb performance according to year of study are presented in Table 4. Mean *S*_*VS*1_ values ranged from 35.6 ± 29.3 points in fifth-year students to 72.5 ± 13.5 points in third-year students. The percentage of time spent outside the prescribed vertical-speed tolerance remained relatively high across all year groups, ranging from 72.7 ± 19.6% to 88.5 ± 8.3%. Considerable within-group variability was observed, particularly in the fourth- and fifth-year groups. The distribution of *S*_*VS*1_ values did not follow a monotonic pattern across year groups, and substantial overlap between individual observations was evident. No statistically significant differences between year-of-study groups were identified for *S*_*VS*1_ (*H* = 6.67, *p* = 0.154, ε^2^ = 0.133, Table 3).

**Table 4:** Vertical-speed performance during climb according to year of study [mean ± SD (min–max)].

Year of study	Time outside tolerance [%] mean ±SD (min–max)	VS RMSE [ft/min] mean ±SD (min–max)	S_VS1_ [points] mean ±SD (min–max)
1st year	84.19 ± 16.58 (59.07–97.12)	544.56 ± 103.85 (375.70–641.54)	72.30 ± 23.07 (33.62–88.64)
2nd year	74.60 ± 10.06 (64.81–87.42)	397.95 ± 143.65 (220.54–576.52)	50.45 ± 21.76 (25.84–78.15)
3rd year	88.53 ± 8.25 (74.36–94.92)	521.23 ± 67.69 (427.22–609.07)	72.48 ± 13.49 (52.77–82.39)
4th year	73.50 ± 12.80 (63.17–89.83)	403.73 ± 198.88 (222.60–722.29)	54.30 ± 29.15 (19.28–92.98)
5th year	72.69 ± 19.61 (45.20–95.07)	292.16 ± 107.68 (139.47–407.78)	35.57 ± 29.27 (0.00–71.04)

Source: Compiled by the authors.

#### 3.1.3 Altitude-hold performance at 4,000 ft

The second flight segment evaluated altitude stabilization during horizontal flight at 4,000 ± 100 ft. Performance was assessed using the percentage of time spent outside the prescribed altitude tolerance range and the RMSE relative to the reference altitude. To enable aggregation of indicators expressed in different units, both variables were transformed into standardized 0–100-point scores using the min–max normalization procedure described in Section 2.6. The resulting altitude-hold score (*S*_*ALT*1_) was calculated as the arithmetic mean of both normalized indicators. Lower *S*_*ALT*1_ values indicate better altitude-control performance.

Equal weighting of both components was applied because no empirical evidence was available to justify differential weighting within the scope of this pilot feasibility study. Consequently, *S*_*ALT*1_ should be regarded as an exploratory composite indicator developed for comparative assessment of altitude-hold performance rather than as a validated operational-performance metric.

Descriptive statistics for altitude-hold performance at 4,000 ft are presented in Table 5. Mean *S*_*ALT*1_ values ranged from 21.9 ± 33.3 points in fourth-year students to 35.5 ± 28.2 points in first-year students. Compared with the climb segment, lower deviation scores were observed across all year groups. However, substantial within-group variability was present in several groups, as reflected by the wide ranges of both time outside tolerance and RMSE values. Differences between year groups were relatively small, and considerable overlap of individual observations was evident. Exploratory group comparisons did not indicate statistically significant differences in *S*_*ALT*1_ performance between year groups (*H* = 2.92, *p* = 0.572, ε^2^ ≈ 0, Table 3).

**Table 5:** Altitude-hold performance at 4,000 ft according to year of study [mean ± SD (min–max)].

Year of study	Time outside tolerance [%] mean ±SD (min–max)	ALT RMSE [ft] mean ±SD (min–max)	S_ALT1_ [points] mean ±SD (min–max)
1st year	7.45 ± 4.40 (0.00–10.93)	60.94 ± 10.56 (37.82–65.97)	35.48 ± 28.18 (7.09–91.05)
2nd year	12.59 ± 13.57 (0.00–32.94)	68.28 ± 47.57 (19.56–148.38)	34.60 ± 36.15 (0.00–91.05)
3rd year	12.05 ± 9.18 (0.00–21.65)	69.68 ± 25.37 (26.70–90.57)	34.46 ± 21.93 (0.55–52.94)
4th year	9.02 ± 16.62 (0.00–39.31)	44.69 ± 28.58 (24.30–99.09)	21.87 ± 33.28 (1.84–79.86)
5th year	10.18 ± 17.15 (0.00–40.12)	57.36 ± 31.58 (36.76–110.99)	27.35 ± 31.98 (6.68–85.49)

Source: Compiled by the authors.

#### 3.1.4 Vertical speed performance during descent

**Figure 2:**
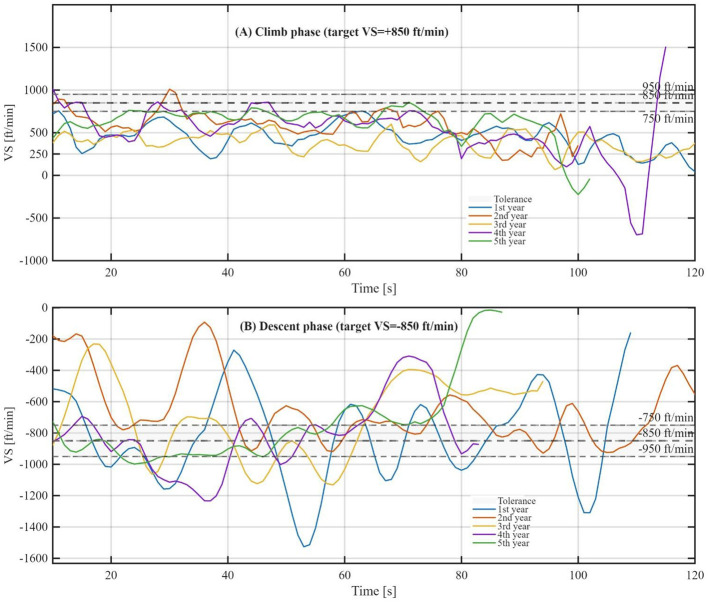
Comparison of vertical speed profiles between year-of-study groups during climb and descent. **(A)** Climb phase with a target vertical speed of +850 ft/min. **(B)** Descent phase with a target vertical speed of −850 ft/min.

The third flight segment corresponded to descent from 4,000 ft to 3,000 ft with a target vertical speed of −850 ± 100 ft/min (Figure 2). Performance was evaluated using the percentage of time spent outside the prescribed tolerance range and the RMSE relative to the reference vertical speed. Both indicators were transformed into standardized 0–100-point scores using the min–max normalization procedure described in Section 2.6. The resulting descent-performance score (*S*_*VS*2_) was calculated as the arithmetic mean of both normalized indicators. Lower *S*_*VS*2_ values indicate better vertical-speed control performance.

Descriptive statistics for descent performance are presented in Table 6. Mean *S*_*VS*2_ values ranged from 32.7 ± 28.4 points in fourth-year students to 72.0 ± 34.1 points in first-year students. The percentage of time spent outside the prescribed vertical-speed tolerance remained relatively high across all year groups, ranging from 68.7 ± 12.7% to 83.3 ± 13.9%. Considerable within-group variability was observed in several groups, particularly among first- and fourth-year students. Although lower mean SVS2 values were observed in the fourth- and fifth-year groups, substantial overlap between individual observations was evident. Among all segment-specific performance indicators, *S*_*VS*2_ was one of only two variables demonstrating a statistically significant overall group effect (*H* = 10.25, *p* = 0.036, ε^2^ = 0.312, Table 3). However, substantial overlap between individual observations remained evident across year groups.

**Table 6:** Vertical-speed performance during descent according to year of study [mean ± SD (min–max)].

Year of study	Time outside tolerance [%] mean ±SD (min–max)	VS RMSE [ft/min] mean ±SD (min–max)	S_VS2_ [points] mean ±SD (min–max)
1st year	83.30 ± 13.88 (59.15–92.31)	595.00 ± 259.95 (210.55–834.63)	71.98 ± 34.10 (14.20–98.86)
2nd year	79.11 ± 4.46 (75.48–86.97)	552.16 ± 119.16 (428.02–715.99)	55.96 ± 23.12 (46.31–72.31)
3rd year	79.99 ± 5.16 (74.58–87.42)	441.94 ± 66.76 (370.38–520.81)	54.10 ± 9.78 (48.50–69.91)
4th year	71.29 ± 15.85 (53.11–83.86)	385.69 ± 163.49 (198.78–611.90)	32.66 ± 28.40 (6.13–72.20)
5th year	68.74 ± 12.72 (47.17–77.97)	368.32 ± 76.51 (276.70–463.37)	37.23 ± 18.68 (6.13–48.51)

Source: Compiled by the authors.

#### 3.1.5 Altitude-hold performance at 3,000 ft

The fourth flight segment evaluated stabilized horizontal flight at 3,000 ± 100 ft. Performance was assessed using the percentage of time spent outside the prescribed altitude tolerance range and the RMSE relative to the reference altitude. Both indicators were transformed into standardized 0–100-point scores using the min–max normalization procedure described in Section 2.6. The resulting altitude-hold score (*S*_*ALT*2_) was calculated as the arithmetic mean of both normalized indicators. Lower *S*_*ALT*2_ values indicate better altitude-control performance.

Descriptive statistics for altitude-hold performance at 3000 ft are presented in Table 7. Mean *S*_*ALT*2_ values ranged from 3.4 ± 5.2 points in fourth-year students to 41.1 ± 29.8 points in first-year students. Compared with the corresponding altitude-hold segment at 4,000 ft, first-, second- and third-year students exhibited higher mean deviation scores at 3000 ft, whereas fourth- and fifth-year students demonstrated lower mean scores. Considerable within-group variability remained present in several groups, particularly among second- and third-year students. Despite differences in group means, substantial overlap between individual observations was observed. *S*_*ALT*2_ also demonstrated a significant overall group effect in the exploratory Kruskal–Wallis analysis (*H* = 10.57, *p* = 0.032, ε^2^ = 0.328, Table 3), although considerable overlap of individual observations was still present.

**Table 7:** Altitude-hold performance at 3,000 ft according to year of study [mean ± SD (min–max)].

Year of study	Time outside tolerance [%] mean ±SD (min–max)	ALT RMSE [ft] mean ±SD (min–max)	S_ALT2_ [points] mean ±SD (min–max)
1st year	23.69 ± 18.33 (8.13–49.66)	113.80 ± 59.84 (57.64–205.76)	41.08 ± 29.84 (18.07–85.05)
2nd year	17.97 ± 30.23 (0.00–70.84)	71.69 ± 72.23 (21.60–196.33)	26.11 ± 37.85 (0.47–97.46)
3rd year	18.59 ± 16.22 (2.81–43.46)	98.09 ± 57.69 (54.37–200.16)	33.97 ± 24.90 (11.26–79.17)
4th year	4.82 ± 7.18 (0.00–17.26)	34.56 ± 15.76 (20.32–77.38)	3.38 ± 5.17 (0.12–13.07)
5th year	4.01 ± 5.49 (0.00–10.65)	38.60 ± 18.31 (19.87–59.39)	7.87 ± 8.91 (0.00–18.02)

Source: Compiled by the authors.

#### 3.1.6 ILS approach performance

The fifth flight segment evaluated approach stability during the ILS Y RWY 27 procedure. The assessed portion extended from the Final Approach Fix to point PK, where pilots were still required to maintain stabilized approach parameters before landing configuration changes.

Performance evaluation combined the percentage of time spent outside the ±100 ft tolerance corridor around the ideal 3° glide path and the RMSE relative to the ideal descent trajectory. Both indicators were transformed into standardized 0–100-point scores using the min–max normalization procedure described in Section 2.6. The resulting approach-performance score (*S*_*APP*_) was calculated as the arithmetic mean of both normalized indicators. Lower *S*_*APP*_ values indicate better approach-tracking performance.

**Figure 3:**
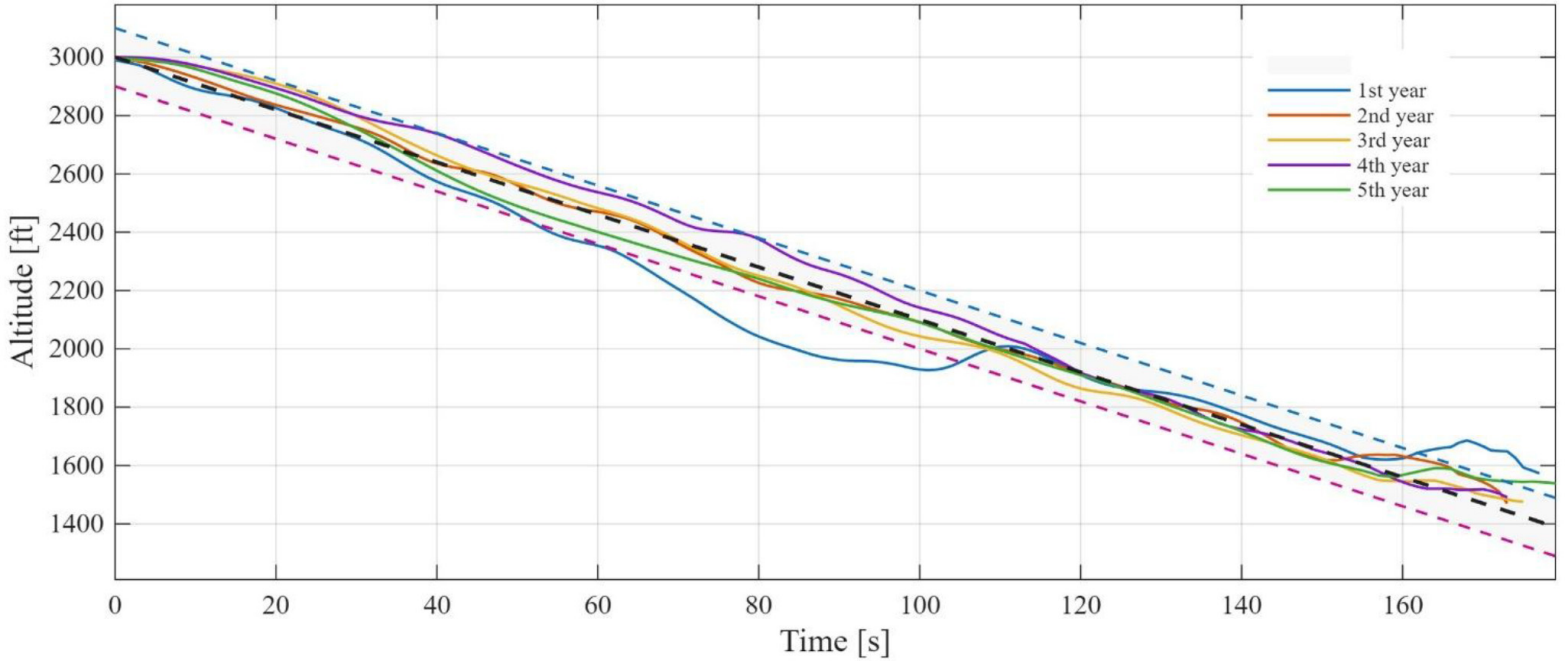
ILS glide path tracking according to year of study. Source: Compiled by the authors.

Descriptive statistics for ILS approach performance are presented in Table 8 and Figure 3. Mean *S*_*APP*_ values ranged from 16.9 ± 9.9 points in third-year students to 38.6 ± 36.7 points in first-year students. Compared with the altitude-hold segments, greater within-group variability was observed in several year groups, particularly among first- and fourth-year students. This variability is reflected by the wide ranges of both glide-path deviations and RMSE values observed within individual groups. Although lower mean *S*_*APP*_ values were observed in the third- and fifth-year groups, substantial overlap between individual observations remained evident. No statistically significant differences between year groups were observed for approach-performance scores (*H* = 2.30, *p* = 0.680, ε^2^ ≈ 0, Table 3).

**Table 8:** ILS approach performance according to year of study [mean ± SD (min–max)].

Year of study	Time outside tolerance [%] mean ±SD (min–max)	ALT RMSE [ft] mean ±SD (min–max)	SAPP [points] mean ±SD (min–max)
1st year	36.33 ± 36.45 (2.70–79.78)	146.59 ± 122.58 (44.79–313.20)	38.56 ± 36.73 (3.83–84.94)
2nd year	25.28 ± 27.95 (0.00–65.77)	80.67 ± 38.54 (30.12–124.93)	21.92 ± 18.96 (0.00–50.50)
3rd year	18.97 ± 14.17 (6.66–42.82)	74.88 ± 12.83 (59.23–93.79)	16.90 ± 9.93 (8.32–33.61)
4th year	22.30 ± 40.33 (0.00–91.60)	97.73 ± 138.19 (33.70–341.04)	24.09 ± 42.14 (0.58–100.00)
5th year	20.32 ± 18.05 (0.76–48.09)	83.83 ± 26.43 (57.85–121.19)	19.73 ± 14.28 (4.87–40.90)

Source: Compiled by the authors.

Across the evaluated flight segments, performance patterns were not uniform. The largest differences between group means were generally observed for airspeed and vertical-speed control, whereas altitude-hold performance demonstrated comparatively smaller between-group differences. Nevertheless, substantial variability and overlap between individual observations were evident across all evaluated performance measures.

#### 3.1.7 Flight experience characteristics

Considerable variability in prior and current flight experience was observed between participants. Although flight experience generally increased with progression through the training programme, substantial differences were present within individual year groups. Several students entered the programme with previous flight experience acquired before university enrolment. Notably, two second-year students reported approximately 150–160 total flight hours, representing some of the highest flight-experience values in the entire sample. Consequently, year of study should be interpreted as an approximate indicator of training progression rather than a direct measure of operational experience.

### 3.2 Overall performance deviation index (PDI)

Lower mean PDI values were generally observed in higher-year groups (Table 9). However, the pattern was not strictly monotonic, as third-year students demonstrated slightly higher mean PDI values than second-year students. Considerable within-group variability was observed in several groups, particularly among first-, second- and fourth-year students, whereas third-year students exhibited comparatively low dispersion of PDI values. Inspection of individual observations revealed substantial overlap between year groups despite differences in group means. The lowest mean PDI values were observed in the fourth- and fifth-year groups, although individual results varied substantially within some groups. Although lower mean PDI values were generally observed in higher-year groups, the exploratory Kruskal–Wallis analysis only approached statistical significance (*H* = 9.19, *p* = 0.057, ε^2^ = 0.259, Table 3).

**Table 9:** Performance deviation Index according to year of study [mean ± SD (min–max)].

Year of study	PDI [points] mean ±SD (min–max)
1st year	52.75 ± 21.28 (26.01–72.44)
2nd year	39.24 ± 22.06 (27.39–78.56)
3rd year	44.33 ± 5.85 (39.33–53.50)
4th year	29.87 ± 18.80 (6.40–52.78)
5th year	25.74 ± 8.99 (12.63–37.73)

Source: Compiled by the authors.

**Figure 4:**
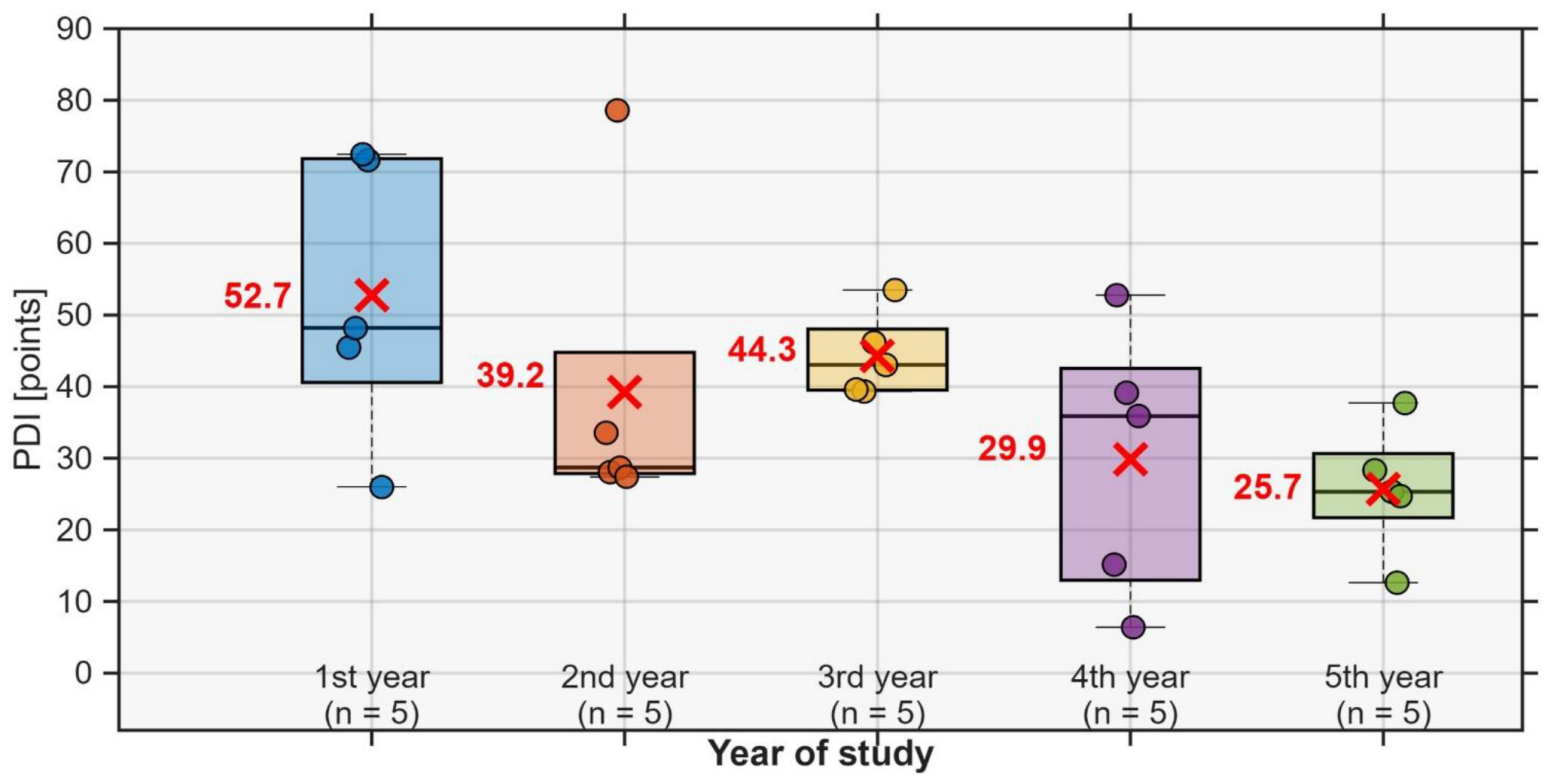
Mean PDI according to year of study. Source: Compiled by the authors.

The distribution of individual PDI values and group-level summary statistics according to year of study are illustrated in Figure 4.

Figure 4 further illustrates the substantial overlap of individual PDI values between year groups and the pronounced within-group variability observed particularly among first-, second-, and fourth-year students.

### 3.3 Physiological response assessment

#### 3.3.1 Heart rate response

Cardiovascular responses during the simulator task demonstrated substantial variability both between and within year-of-study groups. Mean relative heart-rate increase (ΔHR) ranged from 27.1 ± 2.9% in second-year students to 62.5 ± 30.8% in third-year students. Similarly, mean time above the individualized workload threshold ranged from 36.1 ± 9.9% in second-year students to 86.0 ± 27.6% in third-year students.

Table 10 summarizes the descriptive statistics of the evaluated physiological workload indicators. Mean *S*_*HR*_ values ranged from 30.6 ± 6.3 points in second-year students to 72.7 ± 26.4 points in third-year students. Substantial within-group variability was observed in several year groups, particularly among first-, third-, fourth-, and fifth-year students, as reflected by the wide ranges of individual responses. Despite differences in mean *S*_*HR*_ values between year groups, the exploratory Kruskal–Wallis analysis did not identify a statistically significant overall group effect (*H* = 6.25, *p* = 0.181, ε^2^ = 0.113).

**Table 10:** Physiological workload indicators according to year of study [mean ± SD (min–max)].

Year of study	ΔHR [%] mean ±SD (min–max)	Time above threshold [%] mean ±SD (min–max)	S_HR_ [points] mean ±SD (min–max)
1st year	36.0 ± 32.4 (1.0–87.2)	49.6 ± 48.2 (0.0–100.0)	41.7 ± 37.8 (0.0–91.6)
2nd year	27.1 ± 2.9 (22.4–30.1)	36.1 ± 9.9 (23.5–51.0)	30.6 ± 6.3 (22.1–39.5)
3rd year	62.5 ± 30.8 (30.1–104.6)	86.0 ± 27.6 (35.4–100.0)	72.7 ± 26.4 (31.7–100.0)
4th year	39.9 ± 24.2 (24.7–83.6)	53.1 ± 29.1 (20.9–100.0)	45.3 ± 25.9 (21.9–89.8)
5th year	27.8 ± 14.4 (4.2–43.4)	51.6 ± 35.4 (0.0–96.9)	38.7 ± 24.5 (1.5–68.9)

Source: Compiled by the authors.

**Figure 5:**
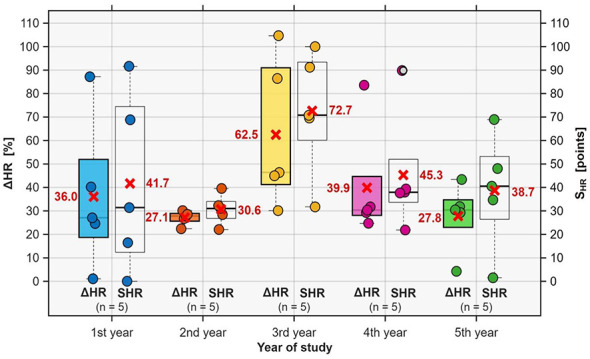
Mean cardiovascular workload response according to year of study. Source: Compiled by the authors.

The distribution of individual observations is illustrated in Figure 5. Considerable overlap between year groups was observed despite differences in group means. Several participants demonstrated markedly elevated cardiovascular activation, whereas others maintained relatively moderate physiological responses under the same simulator conditions.

The distribution of *S*_*HR*_ values did not follow a monotonic pattern across year groups. Third-year students demonstrated the highest mean physiological workload scores. However, this result was influenced by a small number of participants who exhibited particularly high cardiovascular responses during the simulator task. Substantial overlap between individual observations and considerable within-group variability were evident across several year groups. As shown in Table 1, flight experience was unevenly distributed across the sample, and some lower-year students reported flight experience comparable to that of senior students. Furthermore, cardiovascular responses may be affected by individual participant characteristics that were not controlled in the present study. Consequently, differences in physiological activation cannot be attributed solely to year of study.

Figure 5 further illustrates the substantial overlap of individual *S*_*HR*_ values between year groups and the marked variability observed within several groups.

#### 3.3.2 Subjective workload assessment

NASA-TLX subscale scores according to year of study are presented in Table 11. Considerable variability was observed across both workload dimensions and year groups. Mental Demand and Effort generally represented the most prominent contributors to perceived workload, whereas Physical Demand remained comparatively low across all groups. Frustration scores demonstrated substantial inter-individual variability, particularly among first- and fourth-year students.

**Table 11:** NASA-TLX subscale scores according to year of study [mean ± SD].

Year of study	Mental demand	Physical demand	Temporal demand	Performance	Effort	Frustration	RTLX	WWL
1st year	73.0 ± 8.4	28.0 ± 21.7	58.0 ± 22.0	57.0 ± 24.9	72.0 ± 13.0	47.0 ± 29.7	56.0 ± 14.5	61.4 ± 16.1
2nd year	64.0 ± 32.3	18.0 ± 25.6	38.0 ± 24.9	42.0 ± 24.4	48.0 ± 24.6	39.0 ± 35.2	41.6 ± 20.1	49.8 ± 20.4
3rd year	41.0 ± 8.9	32.0 ± 18.2	33.0 ± 16.0	43.0 ± 13.0	64.0 ± 2.2	27.0 ± 16.8	40.0 ± 5.4	45.4 ± 3.2
4th year	65.0 ± 9.4	25.0 ± 27.2	54.0 ± 25.3	46.0 ± 18.5	68.0 ± 5.7	46.0 ± 42.9	50.6 ± 17.1	56.8 ± 18.7
5th year	43.0 ± 11.5	36.0 ± 26.1	45.0 ± 16.6	31.0 ± 23.0	50.0 ± 18.7	47.0 ± 31.5	42.0 ± 10.4	49.2 ± 8.8

Source: Compiled by the authors.

Lower overall workload scores (WWL) were observed among third-year students compared with first- and fourth-year groups. However, this pattern was not consistently reflected across all workload dimensions. For example, third-year students reported relatively low Mental Demand and Frustration scores while maintaining comparatively high Effort ratings. Considerable variability was evident across individual NASA-TLX dimensions and between year groups. The distribution of ratings suggests that perceived workload was shaped by different combinations of subjective workload components rather than by a single dominant dimension.

These findings are consistent with the multidimensional nature of mental workload, which arises from the interaction between task demands, individual characteristics, and available cognitive resources (Hart and Staveland, 1988; Li et al., 2022). Similar to the physiological indicators, substantial overlap between year groups was observed, indicating considerable inter-individual variability in subjective workload perception.

#### 3.3.3 Integrated workload index (WI)

The integrated Workload Index (WI) demonstrated variability in combined psychophysiological workload across year-of-study groups. Mean WI values ranged from 40.2 ± 12.3 points in second-year students to 59.0 ± 14.6 points in third-year students (Table 12). Considerable overlap between groups was observed, and several year groups demonstrated substantial within-group variability, indicating heterogeneous workload responses among individual participants. Similarly, no statistically significant differences were observed between year groups for the integrated Workload Index (*H* = 5.22, *p* = 0.266, ε^2^ = 0.061).

**Table 12:** Workload index according to year of study [mean ± SD (min–max)].

Year of study	WI [points] mean ±SD (Min–Max)
1st year	51.5 ± 21.5 (28.7–83.8)
2nd year	40.2 ± 12.3 (22.5–50.6)
3rd year	59.0 ± 14.6 (36.4–73.0)
4th year	51.1 ± 13.2 (32.8–69.9)
5th year	44.0 ± 15.7 (18.3–58.9)

Source: Compiled by the authors.

The distribution of WI values did not follow a monotonic progression across years of study. Although third-year students exhibited the highest mean WI values, the observed pattern should be interpreted cautiously because workload responses may be influenced by multiple factors beyond academic progression alone, including individual flight experience, prior exposure to flight operations, stress tolerance, and psychophysiological adaptability.

The observed WI values indicate that psychophysiological workload varied substantially among participants and was not consistently associated with year of study. Considerable overlap between year groups and substantial within-group variability were evident across the sample.

**Figure 6:**
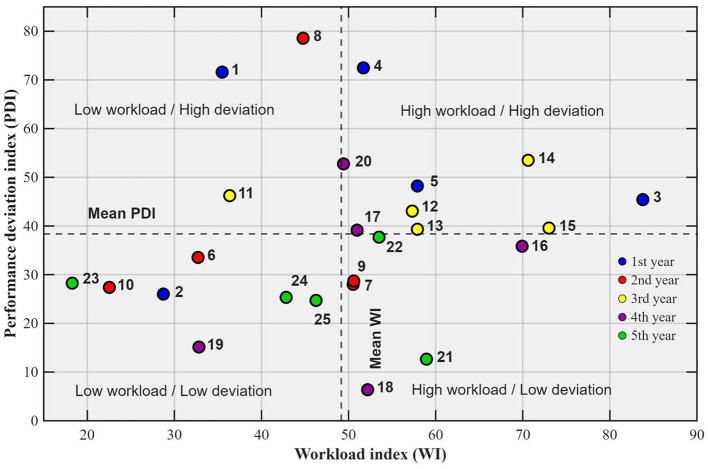
Relationship between WI and PDI. Source: Compiled by the authors.

Figure 6 additionally illustrates the relationship between psychophysiological workload (WI) and operational performance deviation (PDI) at the individual-participant level. Mean WI and mean PDI values divided the dataset into four interpretative quadrants representing different workload–performance profiles. These quadrant classifications are exploratory and should be interpreted as descriptive hypotheses rather than validated categories.

Participants located in the upper-right quadrant demonstrated simultaneously elevated workload and increased operational deviation. In contrast, individuals positioned in the lower-right quadrant demonstrated relatively low operational deviation despite elevated workload levels.

Several third-year students were located in the high workload / high deviation quadrant. However, substantial overlap between year groups was evident, and the limited number of participants within each group precludes broader interpretation of this distribution pattern. Considerable variability was observed across participants, indicating heterogeneous workload and performance responses even among students at a similar stage of training.

Several fifth-year students were positioned in the high workload/low deviation quadrant, indicating relatively low performance deviation despite elevated workload values. Participants located in the lower-left quadrant exhibited both low workload and low operational deviation values.

Considerable inter-individual variability was observed across all years of study, indicating that year of study alone did not fully explain differences in workload responses or operational performance during the simulator task.

#### 3.3.4 Segmental performance distribution

**Figure 7:**
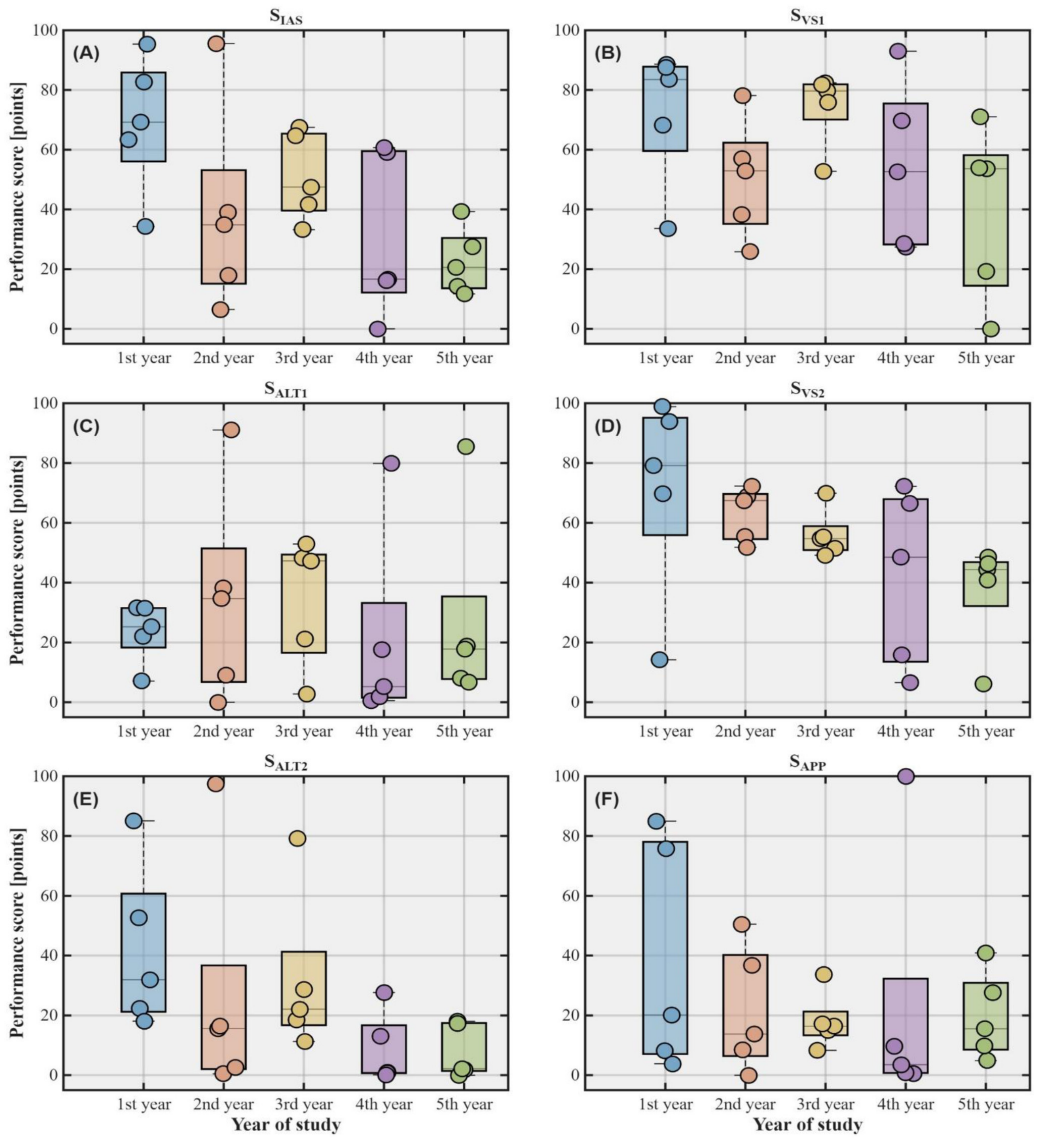
Comparison of performance scores between year-of-study groups for individual flight tasks. **(A)** Indicated airspeed (S_IAS), **(B)** climb vertical speed (S_VS1), **(C)** climb altitude (S_ALT1), **(D)** descent vertical speed (S_VS2), **(E)** descent altitude (S_ALT2), and **(F)** instrument approach (S_APP). Boxes represent the interquartile range, the horizontal line indicates the median, whiskers indicate the range excluding outliers, and individual data points are displayed.

The distribution of segment-specific performance scores according to year of study is presented in Figure 7. Considerable variability was observed both between and within year groups across all evaluated flight segments. The largest dispersion of individual observations was evident for airspeed control (*S*_*IAS*_) and vertical-speed performance during climb and descent (*S*_*VS*1_ and *S*_*VS*2_), whereas altitude-hold performance (*S*_*ALT*1_ and *S*_*ALT*2_) generally exhibited lower deviation scores in several groups.

Substantial overlap between year groups was observed across all performance measures. Although some lower mean performance-deviation scores were evident in selected higher-year groups, the observed patterns were not uniform across all flight segments. These findings further illustrate the heterogeneous nature of operational performance during simulator-based instrument-flight tasks and the considerable inter-individual variability present within the sample.

Figure 7 provides an overview of the distribution of individual observations, group medians, and within-group variability for each segment-specific performance indicator.

### 3.4 Exploratory group comparisons

To further explore whether workload and performance measures differed between year-of-study groups, non-parametric Kruskal–Wallis analyses were performed for the principal workload and performance indicators (Table 3). Significant overall group effects were observed for descent vertical-speed performance (S_VS2_; *H* = 10.25, *p* = 0.036, ε^2^ = 0.312) and altitude-hold performance at 3000 ft (S_ALT2_; *H* = 10.57, *p* = 0.032, ε^2^ = 0.328). Airspeed-control performance (S_IAS_; *H* = 9.36, *p* = 0.053, ε^2^ = 0.268) and the overall Performance Deviation Index (PDI; *H* = 9.19, *p* = 0.057, ε^2^ = 0.259) approached statistical significance. No statistically significant differences were identified for the remaining workload- or performance-related indicators.

Despite the significant overall effects observed for S_VS2_ and S_ALT2_, substantial overlap of individual observations remained evident across year groups. Together with the limited sample size, these findings should be interpreted as exploratory and hypothesis-generating rather than as evidence of definitive training-stage effects. Confirmation in larger cohorts will be necessary to further evaluate the observed patterns and their potential relevance for pilot training and workload adaptation.

## 4 Discussion

The present pilot feasibility study evaluated the applicability of multimodal workload assessment during simulator-based instrument flight tasks in future pilots. Subjective workload perception, physiological activation, and operational performance varied substantially both between and within year-of-study groups. Considerable overlap of individual observations was evident across most evaluated indicators, suggesting that workload responses and flight performance were highly individualized within the present sample. These findings support the concept of workload as a multidimensional construct arising from the interaction of cognitive, physiological and operational demands rather than from a single determinant alone (Hart and Staveland, 1988; Wickens et al., 2021).

Interpretation of differences between year groups should be approached cautiously. Although year of study was used as an indicator of training progression, flight experience was not evenly distributed across the sample. Several participants entered the programme with previous flight-training experience, and two second-year students reported total flight-hour values among the highest observed in the study. Consequently, year of study did not necessarily correspond to operational experience. Recent literature similarly emphasizes that pilot workload and performance are influenced by a combination of expertise, task demands, operational exposure, and individual psychophysiological characteristics rather than by training stage alone (Kyle et al., 2025).

The segment-specific performance analysis demonstrated substantial variability across all evaluated flight parameters. Airspeed control, vertical-speed control, altitude-hold performance, and ILS approach performance all exhibited considerable overlap between year groups and substantial within-group variability. Although lower mean performance-deviation scores were observed in some higher-year groups, the observed patterns were not uniform across all flight segments. These findings indicate that operational performance during simulator-based instrument-flight tasks was influenced by factors extending beyond year of study alone and further highlight the importance of considering individual variability when evaluating pilot performance.

The physiological results demonstrated measurable cardiovascular activation during the simulator task across all year groups. Elevated heart-rate responses and prolonged periods above the individualized workload threshold indicate that even simulator-based instrument-flight procedures may induce substantial psychophysiological activation. These findings are consistent with previous studies reporting associations between demanding flight tasks, increased mental workload, and cardiovascular responses (Mansikka et al., 2016; Wang et al., 2024). However, physiological responses varied substantially between participants exposed to the same flight scenario. Third-year students demonstrated the highest mean physiological workload scores, although this result was influenced by a small number of participants who exhibited particularly high cardiovascular responses during the simulator task. Similar variability in cardiovascular workload responses has been reported in previous aviation studies and may reflect differences in individual characteristics, operational experience, task perception, and psychophysiological responsiveness (Wang et al., 2024; Kyle et al., 2025).

Subjective workload assessment produced a similarly heterogeneous pattern. Mental Demand and Effort generally represented the largest contributors to perceived workload, whereas Physical Demand remained comparatively low across all groups. At the same time, substantial variability was observed across individual NASA-TLX dimensions. Some participants reported relatively moderate subjective workload despite elevated physiological activation, whereas others perceived the task as demanding despite less pronounced cardiovascular responses. Similar discrepancies between subjective workload ratings and physiological indicators have been reported previously, suggesting that these approaches capture related but not identical aspects of workload (Hebbar et al., 2021; Wanyan et al., 2014). This observation reinforces the assumption that subjective workload assessment alone may not fully capture the complexity of psychophysiological stress during flight tasks.

One of the principal methodological contributions of the study was the application of the integrated Workload Index (WI), which combined subjective and physiological workload indicators into a single descriptive measure. Although the index has not been formally validated, it provided a practical framework for exploring psychophysiological workload patterns within the sample. The WI demonstrated substantial inter-individual variability and considerable overlap between year groups, indicating that workload responses were not consistently associated with academic progression alone. Similar inter-individual variability has been reported in simulator-based flight studies, where workload responses were influenced by experience, task complexity, and individual psychophysiological characteristics (Li et al., 2022; Zhou et al., 2024).

The relationship between workload and operational performance was further explored using the WI–PDI framework. The quadrant-based visualization demonstrated that elevated workload was not uniformly associated with greater performance deviation. Some participants exhibited relatively low operational deviation despite high workload values, whereas others demonstrated greater performance deviation despite only moderate workload levels. Previous studies have similarly shown that acceptable operational performance may be maintained despite elevated workload, indicating that performance measures alone may not fully reflect the internal demands experienced by the operator (Hebbar et al., 2021; Luzzani et al., 2024). However, the quadrant classifications in the present study were based on sample means and should therefore be interpreted only as exploratory descriptive categories rather than validated workload-performance profiles.

The substantial overlap observed between year groups further suggests that workload adaptation may not progress uniformly across trainees. Instead, adaptation appears to be influenced by multiple interacting factors, including previous flight experience, individual characteristics, and task-specific demands. Similar conclusions have been reported in recent aviation workload studies, which emphasize that pilot workload cannot be explained solely by experience level or training stage (Li et al., 2022; Wang et al., 2024; Kyle et al., 2025).

From a practical perspective, the study demonstrated the feasibility of implementing multimodal workload assessment within a simulator-based flight-training environment. Continuous physiological monitoring, NASA-TLX administration, and simulator-performance analysis were successfully integrated into a single experimental protocol. All participants completed the experimental procedure, complete physiological, subjective, and simulator-performance datasets were obtained, and no major technical failures occurred during data collection. The successful integration of simulator-performance metrics, physiological monitoring, and subjective workload assessment is consistent with recent recommendations advocating multimodal approaches to workload assessment in aviation research (Masi et al., 2023; Luzzani et al., 2024).

The exploratory group-comparison analyses largely supported the descriptive observations. Statistically significant overall group effects were identified only for descent vertical-speed performance (*S*_*VS*2_) and altitude-hold performance at 3,000 ft (*S*_*ALT*2_), whereas most workload-related indicators and composite measures did not differ significantly between year groups. Airspeed-control performance (*S*_*IAS*_) and the overall Performance Deviation Index (PDI) approached statistical significance. These findings suggest that some aspects of flight-performance control may differ between year-of-study groups within the present sample. Nevertheless, the observed patterns should be interpreted cautiously because of the small sample size and substantial overlap of individual observations.

Several limitations of the present study should nevertheless be acknowledged. First, the experiment was conducted in a simulator environment which, despite standardized and controlled conditions, cannot fully replicate the operational demands of real flight. Real-flight operations involve additional stressors, including operational risk, environmental uncertainty, vibration, acceleration forces, and emotional pressure (Jekl et al., 2025). Second, the study involved a relatively small sample size typical of pilot feasibility studies, limiting statistical power and restricting the generalizability of the findings. The small sample size also limited the statistical power of the exploratory group-comparison analyses, and the absence of statistical significance should therefore not be interpreted as evidence that no differences existed between groups. Third, physiological and subjective indicators may have been influenced by factors that were not formally controlled, including sleep quality, fatigue, circadian influences, physical fitness, caffeine consumption, medication use, emotional state, and individual stress responsiveness. Consequently, heart-rate responses should be interpreted as indicators of overall psychophysiological activation rather than workload-specific responses alone.

Additional limitations include potential cohort effects, inter-individual variability in psychophysiological responsiveness, and the use of year of study as a proxy indicator of training progression. Although year of study broadly reflects advancement within the training programme, it does not fully capture differences in total flight experience, IFR exposure, procedural practice, or operational proficiency. Several participants entered the programme with previous flight-training experience and accumulated flight hours prior to university enrolment, resulting in substantial variability in operational experience within some year groups. Consequently, observed differences between groups may partially reflect unequal flight experience rather than academic progression alone. Future studies should therefore incorporate objective flight-experience indicators, including total flight hours, PIC hours, IFR exposure, and certification level, to better characterize workload adaptation and operational performance.

The sample also contained only two female participants, both belonging to the third-year group. The study was not designed to evaluate sex-related differences in workload responses, and potential influences of sex on physiological workload indicators therefore remain unclear.

Future research should focus on larger longitudinal studies evaluating workload adaptation throughout different stages of pilot training. Repeated measurements across multiple semesters may provide deeper insight into the development of psychophysiological adaptation, operational performance, and workload tolerance. Additional integration of eye tracking, electroencephalography, respiratory monitoring, and machine-learning-based workload prediction methods may further improve the sensitivity and applicability of workload-assessment systems. Future studies should also investigate workload responses during abnormal and emergency flight procedures, where operational demands and decision-making requirements are substantially increased.

## 5 Conclusion

The present pilot feasibility study demonstrated that simulator-based multimodal workload assessment is a feasible approach for collecting subjective, physiological, and simulator-performance data during instrument-flight tasks performed by pilot trainees. The experimental protocol was successfully completed by all participants and enabled continuous acquisition of workload-related and performance-related data under standardized simulator conditions.

The observed results revealed substantial inter-individual variability in subjective workload perception, physiological activation, and operational performance. Considerable overlap was observed between year-of-study groups across most evaluated indicators, suggesting that workload responses and simulator performance were influenced by multiple interacting factors beyond academic progression alone. The findings further support the value of combining NASA-TLX assessment, cardiovascular monitoring, and simulator-performance analysis, as workload may not be sufficiently characterized using isolated subjective, physiological, or performance-based measures.

The study additionally demonstrated the practical feasibility of implementing multimodal workload assessment within a pilot-training environment. However, the present pilot feasibility study was not designed to validate the proposed Workload Index (WI) or Performance Deviation Index (PDI), nor to establish statistically confirmed relationships between workload, flight experience, and operational performance.

Although limited by the pilot feasibility design and relatively small sample size, the study provides a methodological foundation for future research examining psychophysiological workload, operational performance, and workload adaptation during pilot training. Future investigations involving larger samples, longitudinal designs, and more detailed flight-experience metrics will be necessary to evaluate the validity, reliability, and practical applicability of the proposed assessment framework.

## Data Availability

The raw data supporting the conclusions of this article will be made available by the authors, without undue reservation.

## References

[B1] Alaimo A. Esposito A. Orlando C. Simoncini A. (2020). Aircraft pilots workload analysis: heart rate variability objective measures and NASA-task load index subjective evaluation. Aerospace 7:137. doi: 10.3390/aerospace7090137

[B2] Bekesiene S. Smaliukiene R. Vaičaitiene R. BagdŽiuniene D. Kanapeckaite R. Kapustian O. . (2024). Prioritizing competencies for soldier's mental resilience: an application of integrative fuzzy-trapezoidal decision-making trial and evaluation laboratory in updating training program. Front. Psychol. 14:1239481. doi: 10.3389/fpsyg.2023.1239481 38374929 PMC10875136

[B3] Campbell R. D. Bagshaw M. (2008). Human Performance and Limitations in Aviation, 3rd edn. New York: Wiley.

[B4] de Almeida M. F. Soares A. B. F. Campos F. A. D. (2026). Evaluation of workload in military pilots during formation flight instruction: impacts of function and experience. Military Med. 191, e212–e218. doi: 10.1093/milmed/usaf300 40504673

[B5] Hanakova L. Kalivodová M. Vránová K. Hylmar K. Urban D. Socha V. . (2024). “Pilots' physiological responses to specific sound frequencies, in 2024 New Trends in Civil Aviation (NTCA) (Praguez: IEEE), 147–152. doi: 10.23919/NTCA60572.2024.10517849

[B6] Hart S. G. Staveland L. E. (1988). “Development of NASA-TLX (Task Load Index): results of empirical and theoretical research,” in Advances in Psychology (Amsterdam: Elsevier), 139–183. doi: 10.1016/S0166-4115(08)62386-9

[B7] Hebbar P. A. Bhattacharya K. Prabhakar G. Pashilkar A. A. Biswas P. (2021). Correlation between physiological and performance-based metrics to estimate pilots' cognitive workload. Front. Psychol. 12:2021. doi: 10.3389/fpsyg.2021.555446 33959060 PMC8093450

[B8] ICAO (1998). Doc 9683: Human Factors Training Manual. Montréal, QC: International Civil Aviation Organization, 451.

[B9] Jekl J. Jánský J. Dohnal F. (2025). “Multi purpose application of 2SFCA methodology for resource distribution analysis and identification of risks along the frontline,” International conference on military technologies (ICMT), Brno, Czech Republic (Piscataway, NJ: IEEE), 1–8. doi: 10.1109/ICMT65201.2025.11061289

[B10] Kebza V. Šolcová I. (2003). Burnout Syndrome, 2nd edn. Prague: The National Institute of Public Health.

[B11] Kyle A. R. Rouser B. Paul R. C. Jurewicz K. A. (2025). Quantifying pilot performance and mental workload in modern aviation systems: a scoping literature review. Aerospace 12, 626. doi: 10.3390/aerospace12070626

[B12] Lázaro F. L. Nogueira R. P. R. Melicio R. Valério D. Santos L. F. F. M. (2024). Human factors as predictor of fatalities in aviation accidents: a neural network analysis. Appl. Sci. 14:640. doi: 10.3390/app14020640

[B13] Li W. Li R. Xie X. Chang Y. (2022). Evaluating mental workload during multitasking in simulated flight. Brain Behav. 12:e2489. doi: 10.1002/brb3.2489 35290712 PMC9014989

[B14] Luzzani G. Buraioli I. Demarchi D. Guglieri G. (2024). A review of physiological measures for mental workload assessment in aviation: a state-of-the-art review of mental workload physiological assessment methods in human-machine interaction analysis. Aeronaut. J. 128, 928–949. doi: 10.1017/aer.2023.101

[B15] Mansikka H. Virtanen K. Harris D. Simola P. (2016). Fighter pilots' heart rate, heart rate variation and performance during an instrument flight rules proficiency test. Appl. Ergon. 56, 213–219. doi: 10.1016/j.apergo.2016.04.006 27109324

[B16] Masi G. Amprimo G. Ferraris C. Priano L. (2023). Stress and workload assessment in aviation-a narrative review. Sensors 23:3556. doi: 10.3390/s23073556 37050616 PMC10098909

[B17] NASA (2010). NASA TLX: Task Load Index. Moffett Field, CA: National Aeronautics and Space Administration.

[B18] Němec J. (2012). Psychic load as a distinctive and inseperable component of lifeguarding service (Master's thesis). Faculty of Physical Education and Sport, Charles University, Prague, Czech Republic.

[B19] Paas F. Tuovinen J. E. Tabbers H. Van Gerven P. W. M. (2003). Cognitive load measurement as a means to advance cognitive load theory. Educ. Psychologist 38, 63–71. doi: 10.1207/S15326985EP3801_8

[B20] Paas F. G. W. C. Van Merriënboer J. J. G. (1994). Instructional control of cognitive load in the training of complex cognitive tasks. Educ. Psychol. Rev. 6, 351–371. doi: 10.1007/BF02213420

[B21] Parasuraman R. Hilburn B. Molloy R. Singh I. (1991). “Adaptive automation and human performance,” in Effects of Practice on the Benefits and Costs of Automation Shifts, Vol. 3. Final Report. Washington, D.C.: Cognitive Science LAB.

[B22] Shappell S. Wiegmann D. (2000). The Human Factors Analysis and Classification System-HFACS. Oklahoma City, OK: Federal Aviation Administration, 19.

[B23] Smaliukienè R. Bekesiene S. Hoskova-Mayerova S. (2024). Editorial: emotional resilience for wellbeing and employability: the role of learning and training. Front. Psychol. 15:1379696. doi: 10.3389/fpsyg.2024.1379696 38495422 PMC10940539

[B24] Socha L. Liptáková M. Hanák P. Cekan P. Nosek J. Priganc F. . (2020). “Stressful situations in the work of the airport dispatcher,” 2020 New Trends in Civil Aviation (NTCA) (Piscataway, NJ: IEEE), 139–144. doi: 10.23919/NTCA50409.2020.9291017

[B25] Switzer R. Mendez B. H. P. (2025). Management of Sleep and Fatigue in Military Aviation, Library of Congress Public edition. Washington, D.C.: Congressional Research Service.

[B26] The British Psychological Society ed. (2017). Aviation and Aerospace Psychology: Pilot Mental Health and Wellbeing. Leicester: The British PsychologicalSociety.

[B27] Wang P. Houghton R. Majumdar A. (2024). Detecting and predicting pilot mental workload using heart rate variability: a systematic review. Sensors 24:3723. doi: 10.3390/s24123723 38931507 PMC11207491

[B28] Wanyan X. Zhuang D. Zhang H. (2014). Improving pilot mental workload evaluation with combined measures. Bio-Med. Mater. Eng. 24, 2283–2290. doi: 10.3233/BME-141041 25226928

[B29] Wei Z. Zhuang D. Wanyan X. Liu C. Zhuang H. (2014). A model for discrimination and prediction of mental workload of aircraft cockpit display interface. Chin. J. Aeronaut. 27, 1070–1077. doi: 10.1016/j.cja.2014.09.002

[B30] Wickens C. D. Helton W. S. Hollands J. G. Banbury S. (2021). Engineering Psychology and Human Performance, 5th edn. New York: Routledge. doi: 10.4324/9781003177616

[B31] Wingelaar-Jagt Y. Q. Wingelaar T. T. Riedel W. J. Ramaekers J. G. (2021). Fatigue in aviation: safety risks, preventive strategies and pharmacological interventions. Front. Physiol. 12:2021. doi: 10.3389/fphys.2021.712628 34552504 PMC8451537

[B32] Young J. A. (2009). NASA / TM - 2008–*215375 The Effects of Life-Stress on Pilot Performance*. Moffett Field, CA: NASA Ames Research Center.

[B33] Zhou W.-G. Yu P.-P. Wu L.-H. Cao Y.-F. Zhou Y. Yuan J.-J. . (2024). Pilot turning behavior cognitive load analysis in simulated flight. Front. Neurosci. 18:1450416. doi: 10.3389/fnins.2024.1450416 39376543 PMC11456565

